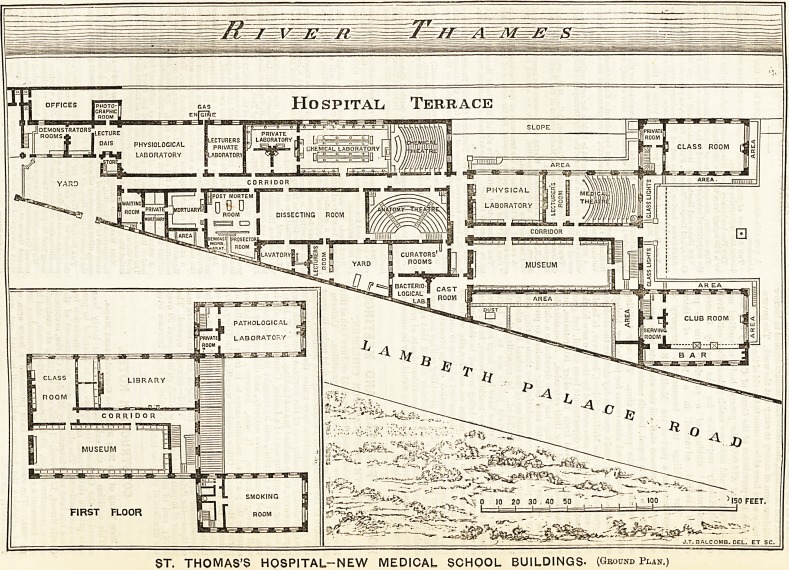# St. Thomas's Hospital

**Published:** 1894-06-23

**Authors:** 


### ST. THOMAS'S HOSPITAL MEDICAL SCHOOL.

### OPENING OF NEW BUILDINGS.

The new Medical Schools of St. Thomas's Hospital, comprise two magnificent laboratories for pathological and biological work. The block facing the river contains the two laboratories, and that facing Lambeth Palace contains on the ground floor a commodious dining-room. This is 16 ft. high, and will seat 100 students at its tables, while a luncheon bar occupies a part of the east recess. At the south end is a small serving room, which is connected to the kitchen and the basement by a lift. A small lift from this floor also will supply the smoking and reading room above with such refreshment as the committee allow in these departments of the club. The entire buildings are fire-proof throughout, the floors being of pitch-pine blocks, laid on Dennet's fire-proof construction concrete; it is heated by both hot water and open fires, and provided with electric light in the sitting-rooms. By the removal of the pathological and biological laboratories from their old quarters additional room is allowed for the chemical and physiological departments, in fact in all the practical workshops of science there is now ample room for present and immediate contingencies. And the students in their leisuretime are accommodated with quarters superior to any other hospital in London, and equal almost to a West-end club.

Mr. G. H. Makins, the Dean, gave the following interesting account of this school: The present medical school buildings were opened in 1871, and since then a very large measure of success has attended the work of the teaching staff. The entry in 1871 amounted to 77 new students, a number largely in excess of any entry for the preceding ten years; in fact, while the school was in temporary premises in the Surrey Gardens during the years 1862-1871 the average yearly entry amounted to only 30. Since the removal of the school to Lambeth, now a period of twenty-three years, 2,167 students have been pursuing their studies here. At the time of the design of the new school the buildings, which were entirely provided by the hospital authorities, were considered to include every requisite for the complete teaching of the various subjects in the medical curriculum, but the steady progress in the direction of making the work of each class essentially practical in nature which has characterised the medical teaching of the last twenty-five years soon rendered changes necessary. The first department to need extension was that of physiology, and immediately on the occupation of the school it was found necessary to re-arrange laboratories originally provided for pathological chemistry for the use of a class of practical physiology. Various minor changes followed, but in the year 1885 the increase in the number of students necessitated further structural alterations. The anatomical department was remodelled and enlarged to its present dimensions, the physiological laboratory was enlarged, and private rooms were added to both departments for the lecturers and demonstrators. In 1892 alterations in the medical curriculum imposed fresh liabilities on our resources, and after prolonged and careful consideration it was decided that, for the continued progress and success of the school, considerable further additions were advisable in order to ensure proper accommodation for the various classes, and to allow each department undivided control over its laboratories and work-rooms. Up to the year 1871 the monetary position of the hospital was such as to allow the governors to provide the buildings needful for one of its most important adjuncts the Medical School, but in 1885 the necessary alterations had to be made at the expense of the teachers, a sum of £2,700 being expended. Again at the present time, as the treasurer has mentioned, the governors, while most anxious to further the

ST. THOMAS'S HOSPITAL—NEW MEDICAL SCHOOL BUILDINGS. (GROUND PLAN.)

welfare of the school, have been unable to provide for extension of the buildings, and hence the teachers decided to borrow a sufficient sum of money to carry out the work. The governing body were here able to come to the aid of the teaching staff, and with the permission of the Court of Chancery they have lent a sum of £16,000, the general body of medical and surgical officers and lecturers making themselves collectively and individually responsible for the yearly interest, and the annual sinking of a sufficient sum to pay off the capital advanced in a period of thirty years. The buildings then become the property of the governors as a part of the Medical School, and the successors to the present staff and the students both present and future will, it is hoped, benefit materially by the action of the teachers of the present time. The new buildings contain laboratories and class-rooms for pathology, biology, and practical surgery, and a spacious day club for the students. The general gain to the Medical School, however, is hardly to be estimated by the area covered by the two new blocks, since not only have new departments been added, but great strain has been removed from the older laboratories, and space has been gained for their extension. A considerable portion of the new buildings has been devoted to a club for the use of the students, the staff being anxious to provide healthy and pleasant accommodation for them during their leisure moments, and the situation of the school has allowed this wish to be fully accomplished. In regarding the club-rooms, however, two important points are to be borne in mind: First, the fact that we have here large rooms well adapted to use as laboratories or teaching rooms, in the event of such further accommodation being needed in the future. The erection of new club rooms on another site would at once set these free, giving an important addition to our teaching capabilities. Second, the erection of the present rooms is an advance in the direction of providing college accommodation for a portion of the students should that appear desirable in the future, since the provision of sets of private rooms on an adjacent site is all that would now be necessary to complete the scheme.

## Figures and Tables

**Figure f1:**